# Increased apoptosis and DNA double-strand breaks in the embryonic mouse brain in response to very low-dose X-rays but not 50 Hz magnetic fields

**DOI:** 10.1098/rsif.2014.0783

**Published:** 2014-11-06

**Authors:** Shreya Saha, Lisa Woodbine, Jackie Haines, Margaret Coster, Nicole Ricket, Lara Barazzuol, Elizabeth Ainsbury, Zenon Sienkiewicz, Penny Jeggo

**Affiliations:** 1 Genome Damage and Stability Centre, Life Sciences, University of Sussex, Brighton BN19RQ, UK; 2 Public Health England Centre for Radiation, Chemical and Environmental Hazards, Chilton, Didcot, Oxford OX11 0RQ, UK

**Keywords:** low-dose radiation, extremely low-frequency electromagnetic fields, apoptosis, DNA double-strand breaks, DNA damage response

## Abstract

The use of X-rays for medical diagnosis is enhancing exposure to low radiation doses. Exposure to extremely low-frequency electromagnetic or magnetic fields is also increasing. Epidemiological studies show consistent associations of childhood leukaemia with exposure to magnetic fields but any causal relationship is unclear*.* A limitation in assessing the consequence of such exposure is the availability of sensitive assays. The embryonic neuronal stem and progenitor cell compartments are radiosensitive tissues. Using sensitive assays, we report a statistically significant increase in DNA double-strand break (DSB) formation and apoptosis in the embryonic neuronal stem cell compartment following *in utero* exposure to 10–200 mGy X-rays. Both endpoints show a linear response. We also show that DSB repair is delayed following exposure to doses below 50 mGy compared with 100 mGy. Thus, we demonstrate *in vivo* consequences of low-dose radiation. In contrast to these impacts, we did not observe any significant induction of DSBs or apoptosis following exposure to 50 Hz magnetic fields (100 or 300 µT). We conclude that any DSB induction by treatment with magnetic fields is lower than following exposure to 10 mGy X-rays. For comparison, certain procedures involving computed tomography scanning are equivalent to 1–5 mGy X-rays.

## 1. Introduction

A highly sensitive *in vivo* system to monitor the cellular response to DNA double-strand breaks (DSBs) has potential application to evaluate risks from exposure to low doses of radiation and to assess potentially genotoxic agents to which populations may be exposed. Mammalian tissues differ in their sensitivity to DNA damaging agents, a feature that has been studied in most depth for ionizing radiation (IR) [1]. The sensitivity of stem cells to DNA damaging agents is particularly important to assess given their potential utility for tissue and cell regeneration. The embryonic neuronal stem and immediate progenitor cell compartments, defined as the ventricular zone/subventricular zone (VZ/SVZ) and intermediate zone (IZ), respectively, are known to be radiosensitive compartments with pyknotic cells being evident after low radiation doses (0.24 Gy) [2–4]. By contrast, apoptosis is rarely activated in the adult brain even following exposure to 10 Gy IR [5,6].

Pioneering studies using the rat brain revealed significant insight into embryonic neuronal development [7]. In mice, cells in the VZ/SVZ, which lies adjacent to the ventricle, undergo rapid replication between embryonic day 8 (E8) and E14, declining by E16.5 [8,9]. At early times during this stage, the VZ cells undergo symmetric division to promote expansion of the stem cell pool; at later times, there is a switch to asymmetric cell division with one daughter entering the post-mitotic, non-dividing IZ compartment and the other daughter remaining as a dividing stem cell [10]. By E16.5, the stem cell compartment largely ceases division. The VZ/SVZ and IZ compartments can be readily distinguished by their position relative to the ventricle [4–6]. Importantly, there appears to be a low threshold level of damage required to activate apoptosis in these regions, which may, in part, be a consequence of the rapid replication [4]. The presence of DNA DSBs, an important lesion that activates apoptosis, can also be sensitively monitored in the embryonic brain using 53BP1 foci analysis [4]. Terminal deoxynucleotidyl transferase-mediated digoxigenin-dUTP-biotin nick-end labelling (TUNEL) represents a sensitive assay to monitor apoptosis, with the VZ/SVZ being more sensitive than the IZ [4,6]. One goal of this study was to define conditions to optimally monitor DSB formation and apoptosis in the developing mouse brain, which we predicted might represent one of the most sensitive tissues responding to DNA damage.

The impact of exposure to low radiation doses has been the subject of substantial recent debate given the increased usage of X-rays and computed tomography (CT) scanning for diagnostic purposes as well as exposure to radiation from flying, and living in areas with higher than average radon [11]. In mouse studies, exposure *in utero* to doses as low as 0.24 Gy either as a single or as a split dose increases the formation of pyknotic cells [2,3]. Substantial analysis of human and animal exposure *in utero* has revealed cognitive impairments following exposure to doses in excess of 0.1 or 0.25 Gy [12–19]. Based on such studies, X-ray exposure during pregnancy is not recommended. However, the response of the embryonic brain to the very low doses incurred during diagnostic X-ray treatments and CT scanning (less than 0.1 Gy) has not been rigorously examined. A second objective, therefore, was to exploit the optimized assays to assess whether such low doses can activate apoptosis and to establish the dose–response relationship.

Having identified an extremely sensitive system to detect X-ray induced damage and the limit to which significant responses can be detected, a third aim was to compare these responses to the effects of exposure to magnetic fields, a ubiquitous physical agent in the current built environment. The impact of exposure to extremely low-frequency electromagnetic fields (ELF-EMF) from power lines and electrical appliances has been the subject of intense debate [20,21]. Epidemiological studies have provided evidence that there is a statistical association between the risk of childhood leukaemia and prolonged magnetic field exposure either due to electrical appliances in the home or due to high voltage power lines proximal to homes at birth [20,22–25]. While it remains uncertain whether this correlation arises from a causal or non-causal relationship between magnetic field exposure and childhood leukaemia, extremely low-frequency magnetic field have been classified as being ‘possibly carcinogenic to humans’ [25]. Biological studies to provide evidence for a potential mechanism have been inconclusive. Most, but not all, laboratory studies have exploited cultured cell lines, and those that focused on possible mutagenic effects have monitored endpoints such as chromosome aberrations, micronucleus formation, mutation induction and single-strand break (SSB) or DSB formation [26]. Although these are potentially sensitive assays, the inherent genomic instability of cultured cell lines and limited growth potential of primary cell lines have provided significant limitations. Possibly as a consequence of this, many of the positive findings reported using such assays have not been reproduced. We reasoned, therefore, that evaluation of the impact of magnetic field exposure in the sensitive *in vivo* assay that we have established would be an important tool to assess risks from such exposure, particularly when evaluated in direct comparison to low-dose X-ray exposure.

We describe the sensitive detection of DSBs and activation of apoptosis following exposure to 10–200 mGy X-rays with a close to linear dose–response relationship for both endpoints. However, we were unable to detect a significant increase in either DSB formation or apoptosis following exposure to 50 Hz magnetic fields.

## 2. Material and methods

### 2.1. Mice

Time-mated pregnant female C57BL/6 mice were obtained from MRC Mary Lyon Centre (Harwell, UK) on E5.5–E6.5. With the exception of experiments to optimize the assays, all animal exposures and preparation of embryonic heads were performed at PHE, Chilton, UK.

### 2.2. Defining the magnetic field exposure condition

Our exposure set-up generated a 50 Hz electromagnetic field where the magnetic flux density was measured and controlled, and the electric field component was negligible. These exposure conditions are similar to many previous studies which described their exposures as being to ELF-EMF. However, we define our exposures as being to magnetic fields because the term EMF does not discriminate whether the electric or magnetic component of the field is dominant.

### 2.3. Exposure to X-rays or 50 Hz magnetic fields

Animals were exposed to X-rays on E13.5, using an A.G.O. HS X-ray System, model CP160/1 (Ago X-Ray Ltd, Martock, UK) at 250 kV constant potential with a compound filter of copper and aluminium (X-ray spectrum with a half-value layer of 2 mm of copper) at a dose rate equal to 4.9 mGy min^−1^. Groups of one to four mice were irradiated in a Correx polythene filtered box (Williton Box Co., Taunton, UK) at approximately 08.30 for 2–20 min to give acute doses of 10, 25, 50, 100 or 200 mGy of X-rays. Following irradiation, animals were returned to the stock holding room. Untreated cage controls were maintained under normal conditions in the same stock holding room.

The magnetic field exposure system for animals was as described previously [27]. Briefly, sinusoidal magnetic fields were generated using a function generator and power amplifier connected to a pair of Helmholtz coils. During exposure (or sham exposure), up to four pregnant mice were housed in a non-metallic cage in the centre of the coils. Animals were provided with standard diet (SDS RM3; Lillico, UK) and water and were observed using a CCTV system.

Animals were exposed to a vertical, 50 Hz magnetic field at 100 µT for 2 h beginning at 07.30 on E13.5 or to a continuous or intermittent (5 min on, 10 min off) field at 300 µT for 15 h beginning at 17.15 on E12.5. During all exposures, the static magnetic field was maintained at 43 µT, the average value of the static field in the laboratory containing the exposure system. Timed sham exposures (no current through the coils) were run on consecutive days and untreated cage controls were maintained in the normal stock holding room. The average background 50 Hz magnetic field in the laboratory housing the exposure system (measured over 24 h using an EMDEX II magnetic field dosimeter) was less than 0.1 µT; average noise levels were 63 dB(A) and ambient light levels were 60 ± 5 lx. Most experiments were performed at Chilton.

### 2.4. Preparation of embryonic heads

At 1 or 6 h after completion of irradiation or magnetic field exposure, the pregnant animals were sacrificed by cervical dislocation and fetuses were immediately removed. The head of each fetus was detached from the body, washed in pre-cooled PBS and embedded using RA Lamb-OCT cryoembedding compound (Thermo Fisher Scientific, Loughborough, UK). The heads were snap frozen in liquid nitrogen-cooled isopentane (VWR, Lutterworth, UK) and stored at −70°C prior to cryosectioning. They were transported to the University of Sussex in dry ice.

### 2.5. Immunohistology

Cryosections (sagittal, 7 µm) were undertaken on a Leica CM1900 cryostat. Before staining, samples were fixed in 3% PFA/2% sucrose/PBS for 10 min and lysed with 0.2% Triton X-100/PBS for 5 min. Immunofluorescence (IF) analysis was performed using anti-53BP1 rabbit polyclonal (1 : 1000; A300-272A; Bethyl), anti-phospho-S10 histone H3 rabbit polyclonal (1 : 200; 06-570; Millipore) and anti-lamin B (1 : 500; SC-6216; Santa Cruz). Secondary antibodies used were Alexa Fluor 594 donkey anti-goat (1 : 500; A11058; Invitrogen) for lamin B and FITC donkey anti-rabbit (1 : 200; 711-095-152; Jackson Laboratories) for 53BP1. Incubation of slides was carried out in a humidified chamber for 30 min at 37°C or 60 min at room temperature. Sections were counterstained with 4,6-diamidino-2-phenylindole (DAPI) (0.05–0.1 μg ml^−1^) and mounted with Vectashield (Vector Laboratories). A Carl Zeiss Axioplan or Nikon eclipse microscope was used for image capturing (SimplePci software). All analysis was conducted blind. Embryos were categorized into groups so that a complete coverage of the exposures (IR or magnetic field, controls or exposed) underwent analysis within a close time frame.

### 2.6. 53BP1 foci analysis and quantification

53BP1 foci were quantified in a delineated region representing approximately half the VZ/SVZ most distal from the ventricle. In trial analysis, we found that the precise position of the boundary chosen did not affect the number of 53BP1 foci scored, as the VZ/SVZ in this region at E13.5 is relatively uniform. Quantification was carried out by scrolling through the entire depth of the section at 100× magnification. The number of cells (identified using lamin B staining) and 53BP1 foci were estimated to allow an estimation of foci numbers per cell. Two sections were quantified for each embryo with a minimum of 60 cells (regardless of DSB numbers) being quantified for each section.

### 2.7. TUNEL staining and quantification

**Figure 1.**
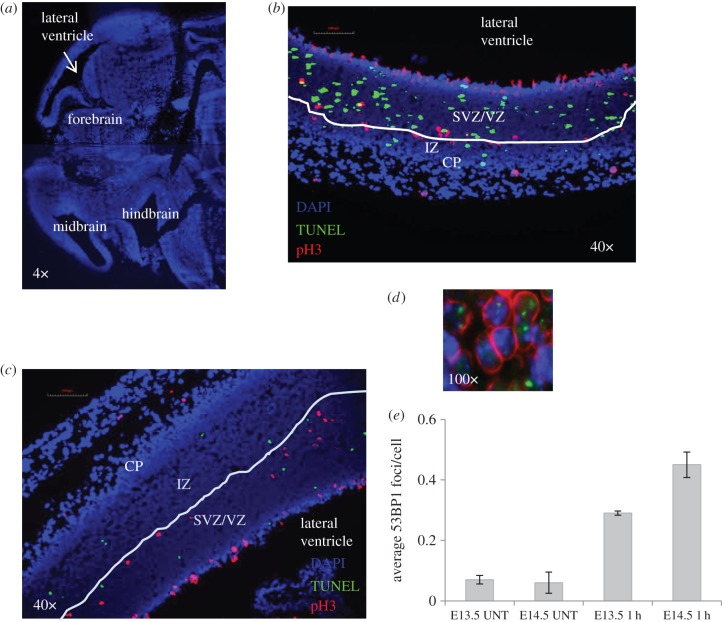
Optimization of the detection of DSBs and apoptosis in the embryonic neuronal compartment. (*a*) An orientation figure to show the region of the embryonic brain undergoing analysis. For orientation, the lateral ventricle is highlighted in subsequent figures. Further details of the region scored for DSB analysis are shown in *c* and *d* and for apoptosis analysis in figure 2*a*. Embryos were exposed to 100 mGy X-rays *in utero* at E13.5 (*b*) or E14.5 (*c*). Mitotic cells were stained using pH3 (red) to delineate the upper boundary between the VZ/SVZ and the IZ. Apoptotic cells are identified by TUNEL staining (green). DAPI staining is shown in blue. The less brightly staining DAPI region encompasses the VZ/SVZ and IZ (the stem and early progenitor cell compartment). The more brightly staining DAPI region represents the cortical plate (CP). The images (40×) show the VZ/SVZ and IZ layers separated by pH3 mitotic cell border, which is highlighted by the white line. At E13.5, the IZ region is very small and the VZ/SVZ occupies the greater part of the less brightly staining DAPI region (*b*). By E14.5, the IZ region has enlarged and the VZ/SVZ and IZ are of similar size (*c*). pH3 staining was not employed for the analysis encompassed in this paper. For 53BP1 foci analysis, an arbitrary line was drawn midway between the ventricle and the CP (brightly staining DAPI region) and 53BP1 foci were enumerated in the half most distal from the ventricle. Trial experiments showed that the precise position of the line did not appreciably affect the number of 53BP1 foci scored. (*d*) Cell delineation using lamin B staining (red) with processing for immunofluorescence using α-53BP1 antibody (green) after exposure to 100 mGy IR. 53BP1 foci were expressed per cell number scored. (*e*) The enumeration of 53BP1 foci at E13.5 or E14.5 in unexposed embryos (UNT) or following exposure to 50 mGy at E13.5 or E14.5.

Quantification of TUNEL-positive cells was carried out in the VZ/SVZ region of E13.5 embryos following *in utero* irradiation. The quantified region is shown in figure 1. TUNEL staining (*In Situ* Cell Death Detection kit, Fluorescein, Roche) was performed according to the manufacturer's instructions. The number of TUNEL-positive cells in the demarcated region was evaluated. The size of the area quantified was assessed using SimplePci software by monitoring the total pixels and estimating the pixels in a defined square area.

### 2.8. Statistical analysis

Statistical analyses were carried out in Minitab v. 15 and Microsoft Excel. The data were tested for adherence to normal statistics using the Anderson–Darling normality test. General linear model analysis of variance (GLM ANOVA) was then applied to test for statistical significance of experimental factors. For the 53BP1 foci IR analyses, the model employed was DSB—Dose (0, 10, 25, 50, 100 mGy), Mother (−3 levels per dose), Embryo (−3; nested within Mother), Sections (2; nested within Embryo); for the DSB EMF analyses, the model employed was DSB—Dose, Mother (greater than or equal to 3 levels per dose), Embryo (3; nested within Mother), Sections (2; nested within Embryo), Intermittency of exposure (intermittent or constant). For the apoptosis IR analyses, the model employed was Apoptosis—Dose (0, 10, 25, 50, 100, 200 mGy), Mother (up to 9 levels per dose), Embryo (3; nested within Mother), Sections (up to 4; nested within Embryo); for the apoptosis EMF analyses, the model employed was Apoptosis—Dose (0, 100, 300 µT), Mother (3 levels per dose), Embryo (3; nested within Mother), Sections (2; nested within Embryo), Intermittency of exposure (intermittent or constant). Individual undertaking the analysis and time point (1 or 6 h) for 53BP1 analysis was also included, where appropriate. *Post hoc* testing with comparisons with the controls dose of 0 Gy (Dunnett's test) and pairwise comparisons (Tukey's test) to compare EMF groups were also carried out, to investigate inter-factor variation between the different dose levels. The data were then fitted to a number of models to investigate linearity of the data and the existence of a threshold, including the Weibull threshold model, a three-parameter log-normal, and a linear no-threshold model. Finally, the fitted model was used to quantify ‘minimum detectable doses’ for DSB formation and apoptosis.

## 3. Results

### 3.1. Optimization of the detection of double-strand break formation and apoptosis in the embryonic neuronal compartment

We have previously observed that apoptosis can be detected after exposure to 100 mGy X-rays in the embryonic neuronal VZ/SVZ compartment of the forebrain at E14.5 [4]. An orientation figure showing the region of the embryonic brain being analysed is shown in figure 1*a*. At E13.5, the VZ/SVZ occupies the major part of the stem and early progenitor compartment of the forebrain (adjacent to the lateral ventricle); by E14.5, the IZ has expanded and the VZ/SVZ represents approximately one-half of the region (figure 1*b,c*). Since we observed that the VZ/SVZ was more sensitive to apoptosis than the IZ, we anticipated that analysis of E13.5 embryos (where most of the forebrain is the VZ/SVZ region) might provide greater sensitivity to monitor apoptosis than E14.5 embryos. The enumeration of γH2AX foci is a commonly exploited assay to monitor DSB formation and repair [28,29]. However, γH2AX foci also arise during replication and are, therefore, observed endogenously in S-phase cultured cells [29]. Consistent with this, we observed a high background of small γH2AX foci in the rapidly replicating VZ/SVZ region, limiting the detection and quantification of radiation-induced γH2AX foci (data not shown). 53BP1 foci represent an alternative DSB marker, which do not form during normal replication. In E14.5 embryos, we observed a gradient of 53BP1 expression extending from low expression in the VZ/SVZ to higher levels in the IZ and as a consequence of the difference in 53BP1 expression, we observed an enhanced ability to detect 53BP1 foci in the IZ compared with the VZ/SVZ [4]. Since the IZ represents a small component of the stem and early progenitor compartment of the forebrain at E13.5, we, therefore, firstly assessed the optimal embryonic stage (i.e. E13.5 versus E14.5) for analysis of DSB formation and apoptosis. At both developmental stages, we quantified 53BP1 foci formation in approximately one-half of the forebrain region, representing the area most distal to the ventricle (as shown in figure 1*b*,*c*); at E13.5 this represents predominantly the VZ/SVZ; at E14.5 it represents predominantly the IZ region (where 53BP1 was more highly expressed). As we observed some variation in total cell numbers in this compartment between embryos (which influenced the results), we stained with lamin B to identify the nuclear membrane and quantified the number of scored cells (figure 1*d*). Thus, 53BP1 foci numbers per cell were quantified. To assess the optimal time for 53BP1 foci analysis, we enumerated foci per cell following exposure to 50 mGy at E13.5 versus E14.5 (figure 1*e*). Consistent with our previous findings, we observed slightly more 53BP1 foci per cell at E14.5 compared with E13.5. Assuming the same level of DSB induction, we conclude that the difference in foci numbers is a consequence of the enhanced 53BP1 expression in the IZ (the predominant area scored in E14.5 embryos), making detection at this stage more efficient.

**Figure 2.**
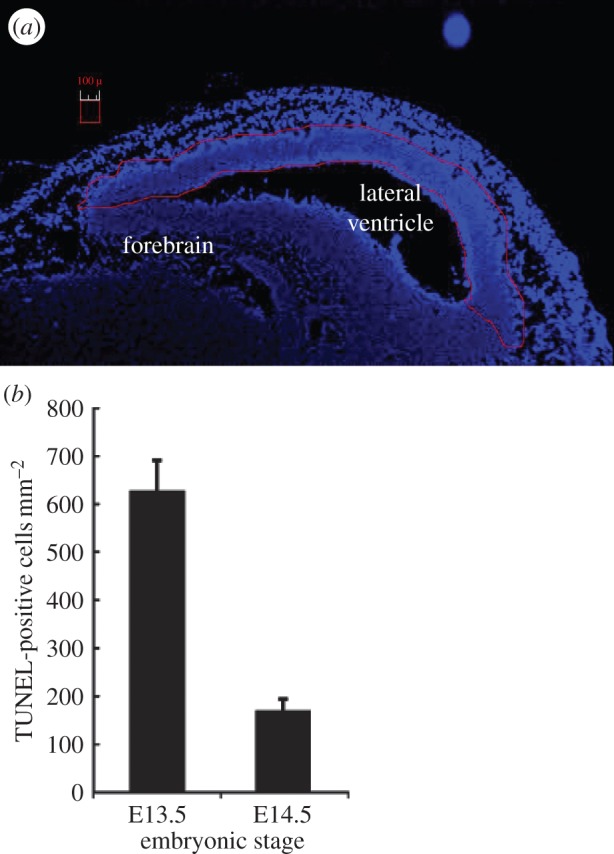
The frequency of apoptotic cells at E13.5 versus E14.5. For analysis of apoptosis, the entire stem and early progenitor compartment of the forebrain was delineated for scoring as marked by the red line in *a*. At this low magnification, apoptotic cells are not visible. (*b*) The frequency of apoptotic cells in the VZ/SVZ after exposure to 100 mGy at E13.5 or E14.5 (scored using higher power magnification). The number of TUNEL-positive cells was estimated after normalizing to the area scored (i.e. TUNEL-positive cells per square millimetre).

Our own and previous studies have shown that TUNEL staining provides a sensitive assay to monitor apoptotic cells in the embryonic brain [2–4,6]. Additionally, we observed that after 100 mGy X-rays, the level of apoptotic cells at E14.5 is maximal at 6 h post exposure [4]. In these previous studies, we delineated the VZ/SVZ from the IZ using the upper mitotic layer as a boundary and/or differentiation markers [4]. However, the staining procedure was not always reproducible. To avoid additional staining procedures, we assessed apoptosis in the entire VZ/SVZ/IZ region adjacent to the ventricle delineated after DAPI staining in E13.5 and E14.5 embryos following exposure to 100 mGy (figure 2*a*). The apoptotic frequency was estimated after normalizing for the area analysed and expressed as the number of TUNEL-positive cells per square millimetre. Thus, in contrast to the DSB analysis, the results are represented per area (square millimetre) rather than per cell. The precise cell number is less significant for quantifying apoptosis than DSBs and the procedure allows a larger area to be monitored for apoptosis, enhancing sensitivity. We observed approximately fourfold greater apoptosis in E13.5 embryos compared with E14.5, which was most likely due to the fact that the delineated region encompasses predominantly the more sensitive VZ/SVZ cells at E13.5 (figure 2*b*). Since there was a substantial benefit in using E13.5 embryos for detecting apoptosis but only a mild decrease in ability to detect DSBs, we employed E13.5 embryos for all subsequent analysis, assessing DSB formation at 1 and 6 h post irradiation and apoptosis at 6 h post irradiation.

### 3.2. Assessment of double-strand break formation and repair after low-dose radiation

**Figure 3.**
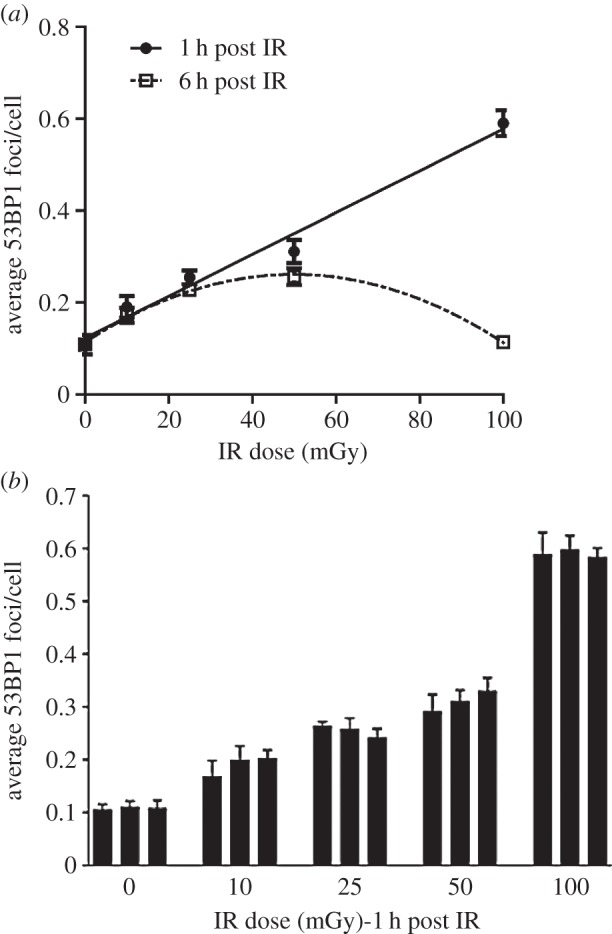
Analysis of 53BP1 foci formation and disappearance in the IZ region after *in utero* exposure of embryos to X-rays. (*a*) Quantification of 53BP1 foci at 1 and 6 h in the IZ compartment following exposure to 10–100 mGy IR. Embryos (E13.5) were exposed *in utero* to X-rays and processed to monitor 53BP1 foci formation at 1 and 6 h post exposure. Results represent the mean of two sections from three embryos from each of three mothers. SEMs are within the data points where not evident. The line represents a linear fit to the data points at 1 h post irradiation. (*b*) The variation in DSB formation for individually irradiated mothers at each dose. Each bar line represents the mean of two sections from each of three embryos derived from a single mother. The errors bars represent the standard deviation. All analysis in this and all figures was carried out blindly.

To assess the induction of 53BP1 foci after low-dose radiation, we quantified 53BP1 foci numbers in a minimum of three embryos derived from each of three mothers (nine embryos) following exposure to 0, 10, 25, 50 and 100 mGy. A minimum of two sections were scored per embryo. The results in figure 3*a* represent the combined analysis of these data; figure 3*b* shows the variation in DSB formation between embryos derived from individual mothers. The number of 53BP1 foci enumerated for each dose for distinct mothers were not statistically distinct, suggesting that there is good uniformity in this response between mothers (*p* = 0.561) as well as between embryos (*p* = 0.942) and sections (*p* = 0.564). We observed a dose-dependent increase in 53BP1 foci following exposure to 10–100 mGy IR with a statistically significant increase being detected after 10 mGy (GLM ANOVA *p* for dose less than 0.001; Dunnett's test *p* for 0 versus 10 mGy less than 0.0001; figure 3*a*). The data were then fitted to a number of potential models. The best fit by far was a linear no-threshold relationship between DSB and dose, with a weighted fit of yield of DSB(53BP1 foci per cell) = 0.123(±0.001) + 0.005(±0.001) × *D* (with dose, *D*, in mGy). The ANOVA *p*-value for the fit was less than 0.001, which indicates a highly statistically significant fit and the *z*-test *p*-values for the individual coefficients were both less than 0.0001. The minimum detectable response based on this fit was then calculated to be approximately 0.233 DSB per cell, which corresponds to approximately 22 mGy.

In addition to assessing 53BP1 foci formation (examined at 1 h post IR), we also enumerated 53BP1 foci at 6 h post IR to assess the level of DSB repair (figure 3*a*).

Interestingly, at 6 h post exposure to 100 mGy, we observed a large reduction in 53BP1 foci per cell, which we take to represent DSB repair, with numbers being similar to those of untreated cells (figure 3*a*). Following exposure to 50, 25 or 10 mGy IR, we observed a greater number of 53BP1 foci remaining with decreasing dose such that after 10 mGy, the number of 53BP1 foci at 6 h post IR was not statistically different to that present at 1 h post IR. Consequently, although the number of 53BP1 foci formed at 1 h was less after the lower doses compared with 100 mGy, the number of foci present at 6 h was greater than that in the 100 mGy irradiated sample at 6 h (figure 3*a*). ANOVA indicates a dose-dependent significant effect of time on the average values of DSBs, i.e. a significant interaction between dose and time (*p* < 0.0001). Pairwise testing demonstrates that the difference between 1 and 6 h is not significant at 10 and 25 mGy (*p* > 0.05), is just significant at 50 mGy (*p* = 0.023) and is highly significant at 100 mGy (*p* < 0.001; figure 3*a*). The potential basis underlying this finding is discussed in Discussion.

### 3.3. Apoptosis increases linearly in the developing brain following exposure to 10–200 mGy X-rays

**Figure 4.**
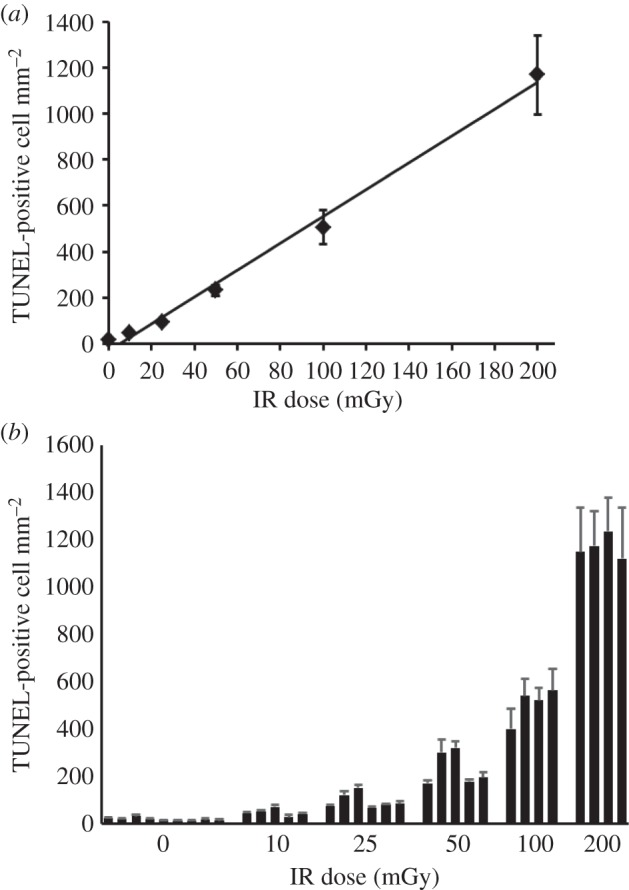
Analysis of apoptosis in the VZ/SVZ region after *in utero* exposure of embryos to X-rays. (*a*) The mean frequency of apoptotic cells in VZ/SVZ from two sections from three embryos from each of three to five mothers. Pregnant mothers were irradiated at E13.5 with the indicated dose and sacrificed 6 h later. The figure shows a linear fit to the data points. (*b*) The level of apoptosis for embryos from individual mothers irradiated at each dose. Each bar line represents the mean of two sections from each of three embryos derived from a single mother. Errors bars represent the standard deviation and are within the data points where not evident.

To assess the level of apoptosis in the VZ/SVZ region after low X-ray doses, we examined a minimum of three embryos derived from each of four to six irradiated mothers at E13.5. A minimum of two sections was analysed for each embryo. For this analysis, we also examined exposure to 200 mGy to enhance the assessment of the shape of the dose–response. Figure 4*a* shows the results for this combined dataset. Figure 4*b* shows the variation in apoptotic induction between embryos derived from individual mothers. Statistical analysis indicated that the variation between embryos from a single mother is similar to the variation between embryos derived from different mothers except at the highest doses (100 and 200 mGy). It is noteworthy, however, that although the variation between mothers and sections was not statistically significant, the variation in activation of apoptosis between embryos derived from distinct mothers was greater than that observed for DSB formation (embryo *p* = 0.074; mother *p* = 0.804; see Discussion for further comments).

GLM ANOVA with *post hoc* testing by Dunnett's test demonstrated a significant effect of dose and a significant difference between all the dose levels and control (unirradiated) embryos with a *p*-value of less than 0.0001. In particular, the sections from embryos exposed to 0 and 10 mGy were significantly different (15.1 ± 1.0 and 38.3 ± 4.5 apoptotic cells mm^−2^ for 0 and 10 mGy, respectively, *p* < 0.001 by *t*-test). There was no significant effect of individual carrying out the scoring (*p* = 0.202) or repeat sections (*p* = 0.271). For testing several different threshold models the best fit was always the linear no-threshold model, with a fit of TUNEL-positive cells mm^−2^ = 5.623(±0.093) × *D* (with dose, *D*, in mGy; Coefficient *p*-value of less than 0.001; ANOVA *p*-value of less than 0.0001). Because the best fit model is a linear no-threshold fit without a constant, and there is a high degree of statistical confidence in the coefficient, the minimum detectable dose corresponding to this fit is approximately 0 (0.002 mGy), corresponding to 1 TUNEL-positive cell. Collectively, these data demonstrate that this represents a highly sensitive assay for detecting the impact of low-dose radiation effects *in vivo*.

### 3.4. Analysis of apoptosis and double-strand break formation in embryos exposed to 50 Hz magnetic fields

**Figure 5.**
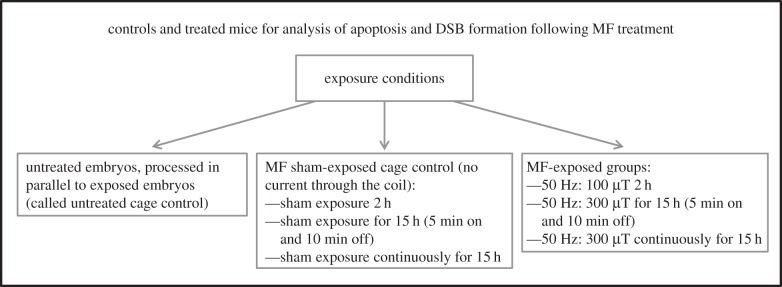
Treatments and controls used for analysis of 50 Hz magnetic field exposure. For all groups, two sections from each of three embryos from each of three mothers were analysed. Embryos were examined for DSB formation (53BP1 foci) and apoptosis as described for the X-ray treatments (MF, magnetic field).

**Figure 6.**
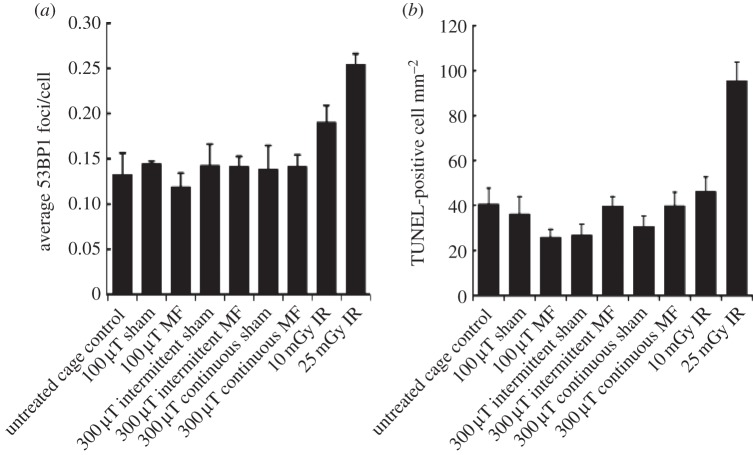
Level of 53BP1 foci and apoptosis following 50 Hz magnetic field exposure. (*a*) Quantification of 53BP1 foci in embryos exposed as presented in figure 5. The control group represents the mean of all the untreated cage control embryos carried out in parallel to the sham or magnetic field-exposed embryos (and are distinct to the controls used in the experiments involving X-ray exposure). The results represent the average of two sections from each of three embryos from each of three mothers (i.e. 18 sections). (*b*) Quantification of apoptosis in the embryos following the treatments shown. Results for X-ray exposure are the same as those shown in figure 4 for the given doses. (MF, magnetic field.)

The optimized assays were next used to assess the effects of magnetic fields on the embryos of pregnant mice exposed using three conditions (figure 5). We included intermittent exposure in our analysis since a previous study reported that intermittent exposure (50 Hz, sinusoidal, 24 h, 1000 µT; 5 min field on; 10 min field off) results in DSB formation assessed by the neutral comet assay [30–32]. We included two sets of controls for each exposure condition: an untreated cage control group was maintained under normal conditions in the stock holding room and a sham-exposed group was placed in the exposure system without the generation of a 50 Hz magnetic field. Sham exposure was conducted on a different day to the magnetic field exposure. Untreated controls were included on the same day as the irradiated animals as well as the sham-exposed animals. As for the analysis above, two sections from each of three embryos from each of three mothers were analysed per treatment. Firstly, we monitored DSB formation at E13.5. The mean frequency of the nine embryos exposed to each treatment is shown in figure 6*a*. There was no evidence of a significant effect of EMF exposure (*p* = 0.100), Intermittency (*p* = 0.503), Mother (*p* = 0.516), Embryo (*p* = 0.304) or Section (*p* = 0.506). All treatment groups were statistically lower than the group exposed to 10 mGy IR (Dunnett's test *p* < 0.05) but were not different from the untreated cage control group or from each other (GLM ANOVA *p* = 0.310). Collectively, this result provides strong evidence that any DSBs induced by exposure to a 50 Hz magnetic field are less than those arising following exposure to 10 mGy X-rays.

Assessment of the mean frequency of apoptosis induction in the different exposure groups is shown in figure 6*b*. There was no evidence of a significant effect of magnetic field (*p* = 0.908), Intermittency (*p* = 0.351) or Section (*p* = 0.879). There was a significant effect of Mother (*p* < 0.001) and Embryo (*p* = 0.018). However, pairwise testing revealed this was due to a significant difference between a few embryos for Mother 1 compared with Mothers 2 and 3 (a total of 9/36 pairwise differences were significant). Pairwise testing also revealed a significantly lower number of TUNEL-positive cells for 100 versus 300 µT (*p* = 0.007). However, as evident in figure 6, the number of TUNEL-positive cells induced by 100 µT was lower than the control values, although the difference was not significant. Surprisingly, the apoptotic frequency of the untreated cage control embryos for the magnetic field experiments was almost double that of the untreated cage controls for the X-ray experiments. The reason for this difference is unclear but reflects the variation in endogenous apoptosis between embryos from individual mothers observed over the extended period of analysis. Notably, however, there was no statistically significant difference in apoptotic frequency for any of the magnetic field-exposed groups and untreated or sham-exposed control embryos (*p* = 0.300) nor the untreated control embryos of the X-ray experiment (Dunnett's test all *p*-values greater than 0.085). There was a statistically significant difference of all the treatment groups compared with those embryos exposed to 25 mGy X-rays (Dunnett's test all *p*-values less than or equal to 0.0002). Thus, we find no evidence that exposure to magnetic fields activates apoptosis with a limit of detection being the level of apoptosis induced by exposure to 25 mGy X-rays. A limitation to the sensitivity of the system is the occasional variation in apoptosis between mothers or embryos as discussed further below.

## 4. Discussion

Previous studies have used γH2AX foci as a sensitive monitor of DSB formation and repair in cultured cells as well as *in vivo* [33,34]. Here, we show that 53BP1 foci can also be used as a sensitive monitor of DSB formation and repair in replicating cells. Previous studies have also shown that the embryonic neuronal stem and early progenitor compartments (VZ/SVZ) sensitively activate apoptosis after exposure to DNA damaging agents [2–4]. Here, we exploit these findings to describe a highly sensitive and reproducible procedure to assess DSB formation and apoptosis after DNA damage in the embryonic neuronal stem cell compartment. A linear fit was observed for doses of 10–100 mGy and, although the minimum detectable dose indicated by the fit was on the order of 25 mGy, detailed factor analysis demonstrated statistically significant DSB formation following 10 mGy. Strikingly, we also show the statistically significant activation of apoptosis after 10 mGy X-rays and observe a dose–response relationship following exposure from 10 to 200 mGy that does not significantly differ from a linear response. The shape of the dose–response curve at low doses of radiation has been long debated and is of relevance for radiation protection. A major limitation is that few assays can monitor impacts of radiation exposure at doses less than 50 mGy *in vivo* or even in cultured cells. While cultured cells provide a tractable system, genetic instability can be enhanced in immortalized cell lines and in primary cells grown in culture. The micronucleus assay can detect the impact of very low doses and for this endpoint, as in our study, a linear response is observed at low doses [35]. Chromatid breakage, a commonly used monitor of radiation exposure, cannot reliably detect damage below 50 mGy [35]. γH2AX analysis is also a sensitive assay, potentially detecting damage after 10 mGy [33]. We suggest that the embryonic neuronal stem and early progenitor cells represent one of the most radiation-sensitive cellular compartments where we can detect not only the initial damage but also apoptosis as a cellular response to it, a finding of significance for considering the impact of low-dose radiation exposure.

The major lethal lesion induced by radiation is a DSB and we have previously observed substantially enhanced radiation-induced apoptosis in a mouse strain deficient in DSB repair [4]. However, it is possible that SSBs, which arise after radiation at a frequency 20-fold greater than DSBs, could also activate apoptosis if they are encountered at the replication fork. SSBs are rapidly and efficiently repaired, however, and in most cell types they do not lead to cell death. It is possible that in the rapidly replicating VZ/SVZ, these lesions are encountered at the replication fork, where they lead to DSB formation and activate apoptosis. However, we favour the notion that the causal lesion is a DSB induced by IR, rather than an SSB generating a DSB following replication. We note that, based on estimations of DSB formation using physical methods, 1 Gy is predicted to yield 25–35 DSBs; thus, following exposure to 10 mGy, only one in three cells are predicted to incur a DSB and few will have two DSBs. This suggests that 1–2 DSBs can activate apoptosis in this cellular compartment. The sensitivity of the system is because a large number of cells can be assayed and a low frequency of affected cells can be detected. Whether the cells that undergo apoptosis represent those that arise in a particular location (or cell cycle position, for example) or whether the results reflect a low-frequency stochastic process is currently unclear. A limitation to the sensitivity of the system is the variation in apoptosis between embryos. We note that this variation appears greater when monitoring apoptosis versus 53BP1 foci formation. The formation of 53BP1 foci is an immediate signalling response to DSB formation and, from a range of studies, appears close to 100% efficient (i.e. 53BP1 foci form at all DSBs). The activation of apoptosis is a more downstream consequence of DSB formation, which may be influenced by a number of factors, including endogenous stress levels. Only a small subset of DSBs activate this response, at least at the time window monitored in our experiments. Importantly, a level of endogenous apoptosis was observed, which provided a limitation to the sensitivity of the analysis. In addition to the analysis of DSB formation and apoptosis, we observed that at low exposure doses (up to 50 mGy), the presence of DSBs was observed for prolonged times compared with cells exposed to 100 mGy. This finding is consistent with a reduced capacity for repair, which has been observed after exposure of non-dividing cultured cells to low radiation doses [36]. Although the inability to lose damage response foci was not observed in lymphocytes exposed *in vivo*, subsequent analysis did reveal a similar phenotype in other tissues [33,37]. It is perhaps significant that the dose below which inefficient repair is observed is 50 mGy, making it possible that cells fail to repair a single DSB. This possibility has been examined previously with evidence suggesting that pre-treatment with H_2_O_2_ can activate a mechanism facilitating the repair of such persisting DSBs [37]. It is also possible that the DSBs are efficiently rejoined but at low doses the damage response foci are inefficiently removed. Another possibility is that low-dose radiation exposure can activate a response, such as a stress response, that itself causes DSB formation. In other words, the persisting DSBs may not be those caused directly by the original radiation exposure. Further work is required to define the basis underlying this important observation.

Having identified a highly sensitive and reproducible system to monitor the impact of radiation exposure, we exploited the assay to examine whether any cellular impact can be detected following exposure to a 50 Hz magnetic field, which is an issue of concern, especially given the association of childhood leukaemia with such exposures. Our exposure set-up generated a sinusoidal 50 Hz magnetic field with a negligible electrical field component. We stress, however, that our exposure conditions are similar to other studies where the conditions are described as representing ELF-EMF exposure. We evaluated exposures to 50 Hz magnetic fields at 100 or 300 μT. Of note, the International Commission on Non-Ionizing Radiation (ICNIRP, 2010) have recommended restricting exposures of members of the public, including pregnant women, to magnetic fields of 200 μT at 50 Hz. Several studies have reported that DSBs can arise after magnetic field (defined as ELF-EMF) exposure [20,25,26,30–32]. However, these studies frequently employ transformed cells, which show inherent genetic instability, and primary cell cultures can have high background damage when nearing the end of their lifespan. The *in vivo* analysis here overcomes these limitations.

Collectively our data provide no evidence that DSBs are induced following 50 Hz magnetic field exposure with a detection sensitivity level equivalent to exposure to 10 mGy X-rays. Furthermore, we were unable to detect any activation of apoptosis after exposure although the sensitivity of detection for this assay is 25 mGy due to variation in the background frequency of apoptosis between mice. We included exposure to intermittent and continuous magnetic field exposure because previously studies had reported that intermittent exposure caused a more significant induction of DSB formation than continuous exposure [31]. However, we observed no significant difference in DSB induction or apoptosis between these two exposures or between untreated or sham-treated cage controls.

In summary, we report a statistically significant increase in DSB formation and apoptosis in the embryonic neuronal stem cell compartment following exposure to 10–200 mGy IR. For both endpoints, we observed a linear response with no evidence for a low-dose threshold. We were unable to detect any significant induction of DSB formation or apoptosis following exposure to 50 Hz magnetic fields. We conclude that any DSB induction by treatment with magnetic fields is lower than that generated following exposure to 10 mGy IR. For comparison, certain procedures involving CT scanning are equivalent to 1–5 mGy X-rays.
